# “Part of getting to where we are is because we have been open to change” integrating community health workers on care teams at ten Ryan White HIV/AIDS program recipient sites

**DOI:** 10.1186/s12889-021-10943-1

**Published:** 2021-05-14

**Authors:** Linda Sprague Martinez, Melissa Davoust, Serena Rajabiun, Allyson Baughman, Sara S. Bachman, Rachel Bowers-Sword, Maria Campos Rojo, Marena Sullivan, Mari-Lynn Drainoni

**Affiliations:** 1 Center for Innovation in Social Work in Health, Boston University School of Social Work, 264 Baystate Road, Boston, MA 02215 USA; 2 Department of Health Law, Policy and Management at Boston University School of Public Health, Boston, USA; 3 Department of Public Health, University of Massachusetts, Lowell, USA; 4 Center for Innovation in Social Work and Health, Boston University School of Social Work, Boston, USA; 5 School of Social Policy and Practice at the University of Pennsylvania, Philadelphia, USA; 6 Boston Medical Center, Boston, USA; 7 Section of Infectious Diseases, School of Medicine & Department of Health Law Policy, School of Public Health, Boston University, Boston, USA; 8 Evans Center for Implementation and Improvement Science, Boston University, Boston, USA

**Keywords:** Community health works, CHW integration, HIV/AIDS care, Implementation strategies

## Abstract

**Background:**

Community Health Workers (CHWs) have long been integrated in the delivery of HIV care in middle- and low-income countries. However, less is known about CHW integration into HIV care teams in the United States (US). To date, US-based CHW integration studies have studies explored integration in the context of primary care and patient-centered medical homes.

There is a need for research related to strategies that promote the successful integration of CHWs into HIV care delivery systems. In 2016, the Health Resources and Services Administration HIV/AIDS Bureau launched a three-year initiative to provide training, technical assistance and evaluation for Ryan White HIV/AIDS Program (RWHAP) recipient sites to integrate CHWs into their multidisciplinary care teams, and in turn strengthen their capacity to reach communities of color and reduce HIV inequities.

**Methods:**

Ten RWHAP sites were selected from across eight states. The multi-site program evaluation included a process evaluation guided by RE-AIM to understand how the organizations integrated CHWs into their care teams. Site team members participated in group interviews to walk-the-process during early implementation and following the program period. Directed content analysis was employed to examine program implementation. Codes developed using implementation strategies outlined in the Expert Recommendations for Implementing Change project were applied to group interviews (*n* = 20).

**Findings:**

Implementation strategies most frequently described by sites were associated with organizational-level adaptations in order to integrate the CHW into the HIV care team. These included revising, defining, and differentiating professional roles and changing organizational policies. Strategies used for implementation, such as network weaving, supervision, and promoting adaptability, were second most commonly cited strategies, followed by training and Technical Assistance strategies.

**Conclusions:**

Wrapped up in the implementation experience of the sites there were some underlying issues that pose challenges for healthcare organizations. Organizational policies and the ability to adapt proved significant in facilitating CHW program implementation. The integration of the CHWs in the delivery of HIV care requires clearly distinguishing their role from the roles of other members of the healthcare delivery team.

## Background

Community health workers (CHWs) have been identified as a critical member of the healthcare workforce. CHW a broad term that includes, promotores de salud, health advocates, peer health advisors, and health navigators among others [1]. According to the American Public Health Association CHWs are “frontline public health public health workers” who have close ties and trusting relationships with the communities they serve [1]. Through relationship development, CHWs build bridges between individual community members and healthcare delivery and human service organizations seeking to serve them [1–3]. In short, the CHW is a boundary spanner with the ability to influence inter-organizational relations [4], linking patients to needed health and social services [2, 3, 5, 6]. This can include engaging patients and connecting them to needed resources as well as improving the healthcare team’s understanding of the ways in which community context influences patient engagement [7]. Through education, advocacy, outreach, case management and navigation, CHWs enhance patient engagement with care, health promoting behaviors and, to some extent, health outcomes [8–12]. Moreover, as a credible health champion, the CHW can enhance community perceptions of the overall healthcare delivery organization, facilitating entry for others in the community by increasing organizational credibility [6].

CHWs have a long history in HIV care [13], particularly in middle- and low-income countries [14–16]. Studies to date indicate that CHWs can improve adherence to antiretroviral therapy [17, 18], psychosocial outcomes [19], and access to social resources that influence treatment seeking, health and well-being [18]. There is a need for more research related to strategies that promote the successful integration of CHWs into HIV care delivery systems. An emerging evidence base exploring CHW integration, has identified both challenges and promising practices. Integration facilitators include clear roles for CHWs and other members of the healthcare team, as well as well as having structured supervision and inclusion in team meetings [20–22]. Of note, these studies explore integration in the context of primary care and patient-centered medical homes. Less is known about CHW integration into HIV care teams in the United States (US).

In 2016, the Health Resources and Services Administration (HRSA), HIV/AIDS Bureau (HAB) launched a three-year initiative, *Improving Access to Care: Using Community Health Workers (CHWs) to Improve Linkage and Retention in HIV Care*, to provide training, technical assistance (TA), and support to Ryan White HIV/AIDS Program (RWHAP) funded provider sites to integrate CHWs into their multidisciplinary care teams, and in turn strengthen their capacity to reach communities of color and reduce HIV inequities. The overall goal of the initiative was to increase the utilization of CHWs to improve access to and retention in healthcare among people living with HIV (PLWH) and to improve health outcomes. Ten RWHAP sites were selected across eight states (NJ, MD, NC, AL, FL, LA, TX, NV) to participate in the initiative.

Sites were provided with extensive technical assistance which included ongoing learning collaboratives, 80 hours of in person and webinar based training, and monthly site coaching calls designed to resolve implementation challenges as well as quarterly supervisor and monthly CHW affinity groups. The training curriculum is publicly available at: https://targethiv.org/library/community-health-workers-hiv-care-curriculum). Trainings were developed based on the Community Health Worker Core Consensus (C3) Project, which defines CHW skills, roles and competencies [23]. The curricula and training program were built by a diverse team of CHWs and supervisors from HIV clinic teams, training and organizational development professionals and HIV content experts in research and practice from across the country. Sites were also provided with an implementation guide https://targethiv.org/library/hiv-chw-program-guide, which was a resource for sites during their implementation, and provided topics that were explored and addressed during the learning collaborative sessions. During sessions sites engaged in activities to facilitate implementation such as developing workflows. Sites also received funding to partially support the salaries of the CHW and supervisor effort as well as their travel to all-site in person meetings. Sites were provided with an implementation guide that contained the core elements for CHW integration. These elements included steps for 1) recruiting, hiring and training CHWs; 2) Establishing a supervision systems including weekly administrative and monthly clinical supervision; 3) identifying and recruiting clients; 4) CHW tasks included strategies for outreaching and engaging clients, weekly contact and support for clients and developing and implementing a client care plan to improve HIV care and treatment; and 5) transition and warm hand off to other members of the care team such as case managers. The overall program was not prescriptive and sites were given flexibility to identify their key client populations served and to integrate CHWs in a way that made sense given their organizational context. RWHAP also decided to partially, but not fully, fund the CHW roles in order to ensure sustainability would be possible post-federal funding. Through the training program and learning sessions, the implementation process was studied and final lessons and adaptations were incorporated in an implementation guide. Outcome assessment [18] and ongoing process evaluation was conducted to examine the CHW integration process across sites. A description of the sites as well as outcomes has been published elsewhere [18].

In this paper, we examine CHW integration into the delivery of HIV care at ten Ryan White HIV/AIDS Program (RWHAP) recipient programs. This paper draws on data from group interviews with the CHW program implementation teams and documents shared with evaluators during site visits. At the time of the interview, the evaluation team engaged site teams in a comprehensive discussion exploring  the steps of implementation as well as the role of the CHW to develop a clear understanding of the process as well as to identify potential facilitators and barriers associated with both implementation and CHW integration [24].

## Methods

The evaluation employed an implementation science conceptual framework, consisting of Pronovost’s 4E Model [25], which is well suited for multi-site projects with centralized support and TA, and the RE-AIM evaluation framework [26, 27]. As specified in the 4E model, site visits were conducted to “walk the process” with teams to better understand the CHW program implementation and contextual factors that served to impede and or facilitate implementation [24]. The evaluation was described in the request for proposals that sites responded to. In addition, sites were provided with a summary of evaluation procedures when they agreed to take part in the project. All sites were expected to take part in the evaluation. While the evaluation was designed by the Boston University team and driven by that team, CHW program sites were actively involved in all aspects of the evaluation. At the initial training before program implementation, significant time was devoted to explaining the evaluation, expected site involvement, reviewing tools for the evaluation and obtaining feedback on the tools and the data collection procedures. This was important in order to ensure that procedures could be adapted to context and would work for the organization. Sites visits were conducted early in the implementation process and at the end of the project period across all ten sites. They involved on-site team group and individual interviews with the evaluation team over the course of a 1–2 day visit.

We draw on group “walk the process” interviews health with site teams for this paper at the beginning of the implementation period and in the last months of the project. Initial site visits occurred approximately 90 days after the launch of the CHW program at each site and accommodated the differential amounts of time programs required to get up and running. Initial site visits occurred between February and July 2018 and follow up site visits occurred between February and July 2019. All protocols were approved by the Boston University Charles River Campus Institutional Review Board (IRB), protocol #4941E and by the Boston University Medical Campus IRB h-36841.

Interviews were scheduled at a time convenient for the CHW site team. At the onset of the visit, informed consent was administered by the evaluators. Each site visit began with an initial group conversation to explain the purpose of the site visit and to get a general sense of how the program worked with all participants present. Participants included CHWs, CHW supervisors, site leadership and site data staff. During interviews, participants reflected on the CHW workflow, what they had planned to do before implementation began, program changes they had made in early implementation, what was going well, challenges to implementation and policies as well as  procedures developed or employed to facilitate implementation. In addition to the team-based group interviews, we also met individually with CHWs and other members of the team. The evaluators met between interviews to debrief on preliminary individual interviews themes and to memo. This was important given power dynamics present in the group interviews. Project documents developed by sites to facilitate implementation were also collected at the time of the interview and facilities were toured to get a better understanding of the CHW work space and clinic workflow. During initial site visits, the evaluation activities were discussed and reviewed again to ensure acceptability and feasibility of the protocol. The site visits lasted approximately 6–8 hours at each site. Each site visit included two evaluation team members, who took detailed handwritten notes during the course of the visit. Team members met following sessions to reflect and debrief about on-site observations. The team then constructed a baseline visit summary.

Follow-up site visits occurred after each program had been in operation for approximately 12 months. At follow-up, we conducted a group interview that addressed where the program was at the time in terms of implementation, lessons learned, adaptations, and how they were thinking about long-term sustainability. Probes were used to encourage team members to explore changes that took place during  the course of implementation, factors that contributed to them, and strategies employed to facilitate implementation. At the follow-up site visit, we also conducted individual interviews with members of the CHW program team. The individual interviews were audio recorded and professionally transcribed. All data were stored on a secure password protected HIPAA compliant drive.

Baseline summaries and follow-up transcripts were uploaded and analyzed in NVivo 12.0 qualitative data management software [28]. For this analysis, deductive codes were developed drawing the on the Expert Recommendations for Implementing Change (ERIC) project [29], which was designed to create a common language to describe implementation strategies by clarifying terms and encouraging their consistent use [30]. A copy of the codes and their respective definitions is available upon request. Two members of the research team coded transcripts using the ERIC codes using directed content analysis [31]. Content in the text illustrating each code was selected and assigned. Memos were written as coders encountered relevant text that did not fit the coding criteria or in cases where there were questions regarding the text. The researchers met after every three interviews to reconcile codes and memos. During each meeting intercoder reliability to determined and areas of disagreement were resolved. A third coder was engaged to discuss discrepancies and overall themes in the data. In cases where relevant themes emerged that did not fit the coding criteria, an inductive code was developed. After coding was completed, reports were generated for each code and summaries were prepared by code. The summaries were discussed by members of the evaluation team to identify the larger narrative within the data. In the final phase of analysis, data excerpts from summaries and illustrative quotes from transcripts were selected to reflect a succinct, cogent written story of the data within and across the identified themes from the data [32].

## Results

For this analysis, we focused on baseline (*n* = 10) and follow-up (n = 10) “walk the process” group interviews with site teams at 10 RWHAP sites. All site team interviews included all CHWs employed by the program, the CHW supervisor, and site director or medical director. In some cases, there were 2–3 CHWs present in the meeting.

The implementation strategies most frequently described by sites were associated with organizational-level adaptations in order to integrate the CHW into the HIV care team. These included revising, defining, and differentiating professional roles and changing organizational policies. Strategies used for implementation, such as network weaving, supervision, and promoting adaptability, were the second most commonly cited strategies, followed by training and TA strategies.

### Role clarification

The most commonly referenced code was the ERIC project organizational change strategy: *Revise Professional Roles,* which was updated to include define and differentiate for the purposes of this analysis. Sites described having to clarify roles to avoid confusion and service duplication:

*… It took a lot of fine tuning to really define specifically how [the CHW role] would complement [other team member roles] without... overlapping too much.*

Ambiguity with respect to role was frequently raised with respect to how traditional HIV case management activities compared to CHW functions. Case management is described as a CHW function in the literature; however, as was noted by multiple sites, the role of “case manager” is specific in the context of HIV care and different from the non-medical case management provided by the CHW. Similarly, sites found it necessary to clarify the difference between the CHW and the peer role. Peers and CHWs both play a supportive role in HIV care. Shared experience provides peers and CHWs with a nuanced understanding of factors the facilitate and pose barriers to care and treatment seeking. The shared experience for peers is a one of a shared diagnosis, and while this may also be the case for the CHW, it is not always the case. The CHW can also share an experience related to community or cultural context. CHWs understand the local landscape and are able to navigate it, serving as a critical bridge between 1) the care team and the community, 2) the patient and healthcare organizations and 3) the patient and the care team. Sites did see a benefit in having the CHW as a peer, because of the ability to bill the Ryan White HIV/AIDS Program for services that fell under the peer role. In addition, CHWs who were peers had a nuanced understanding of treatment and disclosure, which facilitated their ability to build rapport with patients, especially in the case of newly diagnosed patients.

Organizational leadership emphasized having to educate staff about the CHW role before and throughout program implementation. The term CHW, in and of itself, was seen as ambiguous. Sites described it as unclear, reporting it was not descriptive of the role:

*CHW is a vague term and in the agency, we have to get specific with people to help people to see their [CHW] roles.*

In some cases, organizations added words to the title in an attempt to clarify the role. Others reported the Ryan White HIV/AIDS Program HIV care roles did not align with the CHW role and tried to fit the CHW responsibilities into the context of existing Ryan White HIV/AIDS Program-defined roles. The CHW role can be difficult to fund and because it is not reimbursable. In some cases, sites tried to fit the CHW role into an existing title. This was documented by an evaluator during a site visit:

*The site calls the CHWs “Community Health Workers/Support Specialists.” The reason for this is that [name] views them as providing support services, which is different from the case manager role, which in their case is a medical case manager. However, their Ryan White Director, [name], thought it was very important that they maintain the community language in the job title because s/he wants it to be clear that they are not clinic-based only, but go into the community. The language also helps clarify that this staff member is connected to the Ryan White job titles [service categories].*

Sites approached role clarification in a number of different ways. They commonly defined roles in the context of the workflow, outlining relationships between care team members. Task division also helped sites to clarify roles:

*At the start everything was so new, everyone had lots of questions and so we set up the referral processes (where supervisors vetted the request) and that is part of the reason for setting up the referral process. Helped to clarify the role.*

*For a while, CHWs were not assigned to individual patients and they found there was overlap; the team is now working to address boundaries and clarify which worker will be connected to each patient. They have moved from shared clients to individual caseloads, which is considered much better. The model, however, allows for good flexibility and backing each other up. Essentially the [Blinded University Name] CHW program has really helped them to fix an evolving model and gave structure to the CHW role. The goal is for the program to be transitional and last 90 days with people linked to services, but there is flexibility.*

Overall, role clarification highlighted the benefits of the CHW role to the care team, which was seen as contributing to referrals and engagement by team members with the CHW. It also helped to set boundaries around the CHW role, reducing the tendency of providers to “dump” tasks on the CHW. Finally, it reduced territorialism, which occurred when case managers felt the CHW was infringing upon their work and clients.

### Organizational policy and procedural change

The implementation strategy of changing organizational policies was a theme derived from the addition of a new inductive code, *Change Organizational Policies*, which we determined to be a key strategy utilized by sites but not represented by an existing ERIC project strategy. The new code referred to instances where sites changed organizational policies or procedures to accommodate or facilitate implementation of the intervention. Sites described changing policies and standard operating procedures (SOPs) to facilitate CHW integration as outlined in an initial implementation guide provided to the sites. Sites noted developing a number of SOPs related to referrals and workflow, which were instituted to clarify procedures and task coordination across roles. Developing SOPs was more common than changing organizational policies.

*So, we're policy light, and SOP heavy. So, we have procedural pieces that are documented around, with some metrics, not very good metrics, to be quite honest with you. We're working on that now. But we have procedural pieces around, this is how you make a referral, this is who's responsible for what. You make a referral, this is how you close it… so the information, the hand-off in the system happens.*

The most commonly referenced policies were related to working in the field, specifically related to home visiting and transporting clients. Policies associated with home visiting included safety procedures for working in the field:

*I had to put a whole new transportation policy in place with the car logs, the patient logs, and safety – the patient and the staff had to go to the safety classes and what not.*

*… One issue that relates to this program is trying to standardize things across roles such as CHW and have policies and issues that are consistent for people who go into the field, such as policies around home visiting, use of cars, etc.*

In some cases, there were existing policies at the organizational level that needed to be adapted to accommodate the CHW role. There were cases in which sites tried to establish policies at the program level that needed to be adopted at the organizational level. In few cases, organizations were less flexible in accommodating the CHW role and fieldwork outside of the organization, which limited the role of the CHW. In these instances, CHWs were unable to conduct home visits or to meet with patients in the community or to accompany them to appointments at community-based agencies. They instead coordinated with patients and community service providers by phone and text messaging. Issues of safety and liability were raised in discussions related to home visiting policies.

*… one of the things we're implementing now that we didn't have before was the use of home visits. Safety issues and things like that. But now we're talking about, okay, if we begin to allow home visits, what will that look like? What do we need to do to make sure she [CHW] stays safe?...*

Of note, for some CHWs at sites that were not open to considering home visits due to safety, CHWs described some frustration, particularly when the “unsafe” areas were their communities.

Sites also referenced human resource (HR) and labor related policy changes relevant for the CHW program implementation. It was important for sites to look at HR policies related to hiring and degree requirements. In some cases, a CHW position’s educational requirements needed to be changed, such as the previous requirement of a college degree. In others, all positions required a driver’s license, but this was changed because the CHW, like many clients, navigated the community on the bus. Sites also described needing to look at policies related to work hours to accommodate the CHW role. This included policies like flexing the CHWs’ schedule so they could work more when clients were available (e.g., evenings), and policies that allowed CHWs to work more flexible hours compared to other staff.

### Network weaving

Sites used the implementation strategy of *Promote Network Weaving,* which the ERIC project describes as building on existing high-quality working relationships and networks within and outside the organization to promote information sharing and collaborative problem-solving, to facilitate CHW integration. Network weaving allowed CHWs to strengthen relationships both within and outside of the organization. Internal network weaving operated through referral mechanisms, care team co-location, care team huddles, and personal relationship building as well as communication that occurred through the electronic medical record between CHWs and other care team members. Internal network weaving amongst the care team improved CHW-client interactions, helped CHWs in their professional development (e.g. supervision), and self-care:

*Well, now we're partnering. We have now an intake committee meeting to address the initial intake, the first impression, and she's [the CHW] part of that group because it's essential that she keeps the people linked, especially if she's tested them in the field. They already know her. We have DOH. They cannot always approach our clients appropriately and she is the bridge for that. So, she's very involved with the day to day medical care. And as a referral, she gets flags all the time from staff, "I think that this is someone that [you should see]. It would help if you could reach out to him. I think this is a person who would benefit from your services.” So, she's part of the holistic picture from the beginning.*

*In addition to the daily huddle, they have other meetings. There is regular case conferencing, done together with both the administrative and clinical supervisors. There used to be case review meetings … . There is also a monthly sexual health team meeting and a wellness meeting at another HIV agency that the CHWs attend. There is an annual [agency] half-day clinical services meeting and a monthly case conference meeting about Spanish-speaking clients.*

CHWs performed a great deal of internal network weaving in their role, particularly as a liaison between providers and clients. Network weaving also enhanced role clarity and improved care team cohesion.

In some organizations, particularly hospitals or health centers, there was not as much evidence of internal network weaving. When it was present, it was from CHWs engaging in informal network weaving with co-workers through the development of positive working relationships, which allowed them to find the right people to ask questions of and get things done for their clients outside of the formal organizational structures:

*There are no other regular meetings in which she [CHW] has participated. The providers have monthly meetings at the [name] site. The staff report that it is very difficult to get on the agenda for the provider meeting. There has also been discussion of working with the VP of nursing to have a care coordination meeting (with Medical Assistants). … The care team does not participate in huddles either. As soon as the providers arrive at the clinic, they start seeing patients.*

In this case, formal internal network weaving was more difficult to achieve. The healthcare delivery system was large with variety of specialized sub-groups. Not having formal network weaving opportunities hindered integration. The CHW had to seek out ways to develop a network, engage with team members and develop relationships independently.

External network weaving was utilized in the context of building a positive image of the clinic in the community, connecting clients to external resources and assistance, and facilitating communication between different local care settings and community-based organizations (CBOs).

*… [The CHW] is aware of so many more resources than perhaps somebody who would be stuck in a building 40 hours a week.*

*… We want them to link outside of us. We never want to think of [our agency] as the only resource a patient knows. We want them to know and utilize [outside agency], which has housing assistance, has bus pass assistance, has all the other stuff that we don't have, and we can refer them to [another outside agency], if needed, which is another CBO that that has other services.*

*Well, I usually just work with the HIV program. The other community health worker, she actually is housed in the office I'm housed in. So, I see her from time to time, she is out and about a lot too … She is trying to connect me with other agencies as well. Agencies that she's already connected with. So, that has been helpful and so we bounce ideas off of each other about community activities that's happening or agencies that have questions about stuff. It's easy to refer people, agencies to me, just so I can get I there and share the information that I know.*

External network weaving also contributed to professional identity development and role clarity for CHWs, enabling them to connect with other CHWs through local and national professional organizations and build professional skills.

*I would have liked to be connected to the Ryan White Council sooner too. So, I think that has been really useful to understand HIV in Houston as a whole and Ryan White as a whole. So, I'm on one of the committees and I did their Project Leap, which is a 17-week training course.*

*… last year, right around this time, we were invited to present at the AETC Regional Conference. And we got to do a presentation, and [CHW name] spoke on how we were integrating a CHW in HIV care.*

Some CHWs had personal relationships with individuals working for other local care settings or CBOs so they could facilitate connections for their clients. Some noted working closely with the housing programs in the area and the city’s housing authority. Sometimes communication occurred between organizations; for example, one clinic had a pharmacy that would contact them when a client was not picking up their medication, which would trigger a call from a CHW to the client. External network weaving occurred in terms of other local stakeholders (e.g. the county health department) learning that a CHW was working out in the community and could help them facilitate some of their own activities with clients (e.g. reporting and informing partners). CHWs sometimes attended events hosted by other local stakeholders.

### Supervision

Sites also referred to the ERIC project implementation strategy of *Provide Clinical Supervision*, which was updated to encompass administrative supervision as well. Sites generally conducted formal supervision meetings with CHWs at least once a week, but most supervisors were also in contact with CHWs on a daily basis through informal interactions. Some sites held monthly group supervision meetings. Open and comfortable communication between CHWs and supervisors was seen as positive, while micro-managing was seen as negative. Interactions were usually more related to administrative, rather than clinical, supervision. Not all sites viewed the clinical supervision as necessary or helpful, while almost all of the sites had administrative supervision.

Sites varied in how they perceived the importance of clinical supervision at the onset of the project. CHWs appreciated having clinical supervision. Clinical supervision most often referred to work with a social worker or other behavioral health provider, and addressing mental health concerns appeared to be the most common topic of supervision meetings. However, team members at one site noted how clinical supervision could encompass training and supervision in both mental and physical healthcare. They noted that while their CHWs were not involved in clinical practice, their involvement with the care team meant they were often learning about clinical aspects of clients’ treatment for HIV and other chronic conditions. At another site, the team members did not have formal clinical supervision, which leadership perceived as unnecessary based on the CHW’s role in their organization. However, the CHW did have access to an MSW for supervision if needed.

### Adaptability

The ERIC project implementation strategy of *Promote Adaptability* also featured prominently in this analysis. Sites found they needed to be adaptable both in implementation of the CHW programs and in response to external changes. A common adaptation was making changes in eligibility criteria and the population of focus for the CHW program, usually to widen the scope of the program. Making changes to the referral process in order to facilitate communication and coordination amongst care team members was also common:

*I think it's we're learning as we're going, … it's kind of hard to say what we would do differently until we've actually done it. It's like, "We should have done that differently." But in hindsight, I think we did a fairly good job. Of course, there were lessons learned, and things that we could tweak, but I think part of getting to where we're at now, is because we've changed. … We were able to be fluid. So, I think definitely that would be something to keep, being able to be fluid. … I think that because we do remain flexible, like even sharing with you today, Dr. [Name] had a patient and he wanted someone to talk to him and the nurses already working the schedule, and so someone called me and said, "Will you come down and talk to the patient." So, I think that that being flexible is a prerequisite for everything that we try to do around here to keep a balance.*

One site was exploring the use of virtual encounters for CHWs and clients. They noted this could help CHWs reach clients when they have barriers to coming in to the clinic, such as the need for child care. They suggested adapting to virtual encounters could help with program sustainability. Being adaptable in terms of policy changes was also key, as previously discussed. Sites found that program length did not work for all clients. This was perhaps the most common example of adaptability, in that sites moved toward flexible program lengths as their programs evolved, and the length of time clients spent in the CHW program was dependent on their individual needs. Finally, CHWs themselves emphasized the importance of being adaptable and flexible in their role. At some sites, the role of the CHW within the wider care team evolved over time:

*Flexibility allows for urgent things to be addressed - say if a client is in the clinic and there is an urgent need.*

### Training and technical assistance (TA)

A variety of ERIC project strategies related to training and TA for stakeholders were discussed by sites, such as *Distribute Educational Materials*, *Conduct Ongoing Training*, and *Create a Learning Collaborative*. In particular, training was an important implementation strategy for CHW integration. Sites received ongoing dynamic training from a centralized TA team at Boston University and participated in a learning collaborative across the 10 sites. Educational materials that site team members received through the learning collaborative and centralized trainings were subsequently shared with other site staff and site leadership. They also enjoyed the training for the most part, finding it beneficial with respect to the information and materials provided and the relationships developed through the process:

*I met some great people. We learned different ways of doing things. Working with other teams and other places gave us a bigger outlook of what was possible, what we could do. What we maybe need [ed] to [tweak] or do differently. It showed us that we weren't the only ones …*

There was some variation with sites feeling as though the training could have been handled in-house. Meanwhile, others expressed some components could have been online. These issues speak to the challenges of making training relevant for sites with differing levels of infrastructure and experience. Sites appreciated sessions where they could learn from one another over those that were content heavy. The training format provided an opportunity to network work with and learn from peers across the country.

## Discussion

Sites integrating CHWs into their HIV care team described using a number of implementation strategies. Site team interviews revealed implementation strategies focused primarily on revising, defining and differentiating professional roles; changing organizational policies; promoting network weaving; providing supervision; promoting adaptability; and engaging with different training and technical assistance strategies. These findings are consistent with the literature [22]. Payne et al. (2017) evaluated CHW integration in 24 organizations’ healthcare delivery organizations. They, too, found role confusion and ambiguity posed challenges to CHW integration [21]. This was a particularly salient issue when other health professionals could not see the value added by the CHW [21]. Similar to our findings, Payne et al. (2017) found providers with limited time and high caseloads saw benefits in working with CHWs who were able to address non-medical needs. When CHWs were seen as facilitating provider ability to work with patients, integration was improved [21].

Although our findings mirror those of Payne, et al. (2017), we did note added challenges associated with implementing CHWs in HIV care. In general, case management is characterized as a CHW role [23]. However, Ryan White HIV/AIDS programs provide for a specific case manager role. This context highlights the importance of distinguishing the CHW case management function from the role of the medical case manager in HIV specifically. For example, case managers provided medical case management, whereas the CHW was able to provide non-medical case management that addressed social factors which impact disease management, such as transportation, housing and food security. In fact, we found that the CHW and case manager roles in HIV care could be quite complementary. The size of the case manager patient panel was generally too large to allow for the intensive case management focused on social factors, which CHWs offered patients. Moreover, medical case managers were more likely to see patients in the office. Fulfilling their role, CHWs were able to, in most cases, bridge the office and the community. The CHW role allowed for more time to provide more intensive assistance, such as helping patients complete important paperwork and program applications, as well as to accompany them to appointments in the community. Thus, the CHW was able to provide help with the continuity of care by bridging the clinic and the community and coordinating with the case manager.

Like others, we found the promoting network weaving was critical to integration. Allen, et al. (2015) conducted a mixed methods study to explore their CHW perceptions of integration of healthcare integration. CHWs from across the country (*n* = 265) were surveyed and 23 of those surveyed then took part in semi-structured interviews exploring organizational characteristics that facilitate their work as a CHW. Internal network weaving through meeting participation was important in facilitating co-learning and role clarification [20]. In addition, meetings served as a critical forum for communicating factors associated with patient care [20]. External network weaving was identified as supporting CHW’s work with connecting clients to needed resources. Like others, we found it also supported professional identify development as CHWs built their networks in the broader community through connections with agencies, other CHWs, and professional organizations [20].

Site teams found information provided in the implementation guide as critical to informing their approach to role definition. In addition to content-based training to promote client adherence to HIV treatment, CHWs and supervisors received training in strategies to ensure role clarification, working as part of team, trauma-informed care, advocacy, communication and conflict resolution and professional development (training curriculum and materials can be found at: https://targethiv.org/library/community-health-workers-hiv-care-curriculum). This training and supervision approach coupled with coaching and peer to peer learning collaboratives supported program implementation. Sites used materials provided in the implementation guide to create role descriptions, and subsequently held meetings with staff both prior to and during implementation to review these role descriptions with their teams. Through our learning sessions and feedback from CHWs and team member revisions were made to the guide based on practice and is available at: https://targethiv.org/library/hiv-chw-program-guide. A recent study from the National Academy of Medicine found that supportive supervision strategies that encourage and engage CHWs and supervisors in a process that promotes learning, safety and professional development are critical for CHW effectiveness in the healthcare settings [33].

Organizational policies and procedures proved significant in facilitating CHW work. There are few health professionals that span healthcare delivery organizations and the community; in the case of CHWs, there has been a shift in workforce integration from community partners and informal resources to direct hires [34], which calls for organizations to reassess policies and practices that govern the CHW role. Internally, SOPs facilitated role clarification, while policies governing working in the community such as transportation practices and home-visiting facilitated resource referrals and access to services.

We found that, wrapped up in the implementation experience of the sites, there were some underlying issues that may pose challenges for healthcare organizations. More specifically, implementation highlighted the ways in which structural oppression and power play out in healthcare delivery systems. Policies related to home-visiting and transportation might represent organizational values about whether and how to build more authentic bridges to populations that experience healthcare inequities. Meanwhile, resistance to policies that facilitate the ability of CHWs to navigate the community, couched in the language of liability and safety may represent underlying bias and stereotypes held by institutions about communities of color.

Traditionally, CHWs have been housed in CBOs and grassroots organizations in the community [34], and able to freely navigate the local landscape. Their integration into healthcare delivery organizations poses new challenges. Beyond home visiting policies, hiring can also pose a challenge. Human resource policies should be considered to ensure diverse educational backgrounds, such as a high school equivalency (GED) or high school diploma, are adjusted to reflect the typical characteristics of CHWs. This is critical because CHWs are hired credibility in the community and the trust relationships they have with community members not because of their educational credentials. As such, policies related to CHW educational requirements posed initial challenges for some sites. Traditionally, professionalization has been associated with higher education, yet this form of education is not a predictor of successful patient engagement. Indigenous form of knowing are often overlooked or dismissed, however, they can be critical for patient engagement and the establishment of trust. This raises the question of how organizations are going to value different - less institutionalized - ways of knowing, in an equitable way. This also has implications for compensation policies that may be tied to educational attainment, which may not be appropriate for CHWs and inadvertently underestimate their value and contribute to salary inequity. These discussions highlight important questions about equity - are organizations ready to elevate the role of individuals who are highly knowledgeable, but not in the way institutions and organizations have typically assessed knowledge? Indeed, the integration of the CHW role may present an occasion for healthcare delivery organizations to reassess policies that may unintentionally marginalize CHWs limiting both career mobility and patient engagement.

This work is not without limitations. With on-site data collection only occurring twice during CHW program implementation, there may have been elements of implementation not captured. If data from monthly calls had also been analyzed, additional themes may have emerged. Similarly, as noted, we drew on the group interviews with site teams and not individual CHW interviews. Although the group interview was informed by individual interviews and included CHW perspectives, drawing on both data sets may have provided more nuance related to the role of the CHW and barriers to implementation.

Using the codes from the ERIC project may not have allowed us to pick up as much nuance from the data. However, we added the inductive codes and analysis for this reason. Sites emphasis on the importance of changing organizational policies may indicate an implementation strategy that could be incorporated into the ERIC project’s list. Some implementation strategy categories were not discussed by the site team members, such as those related to planning and assessment, but this was likely due to a lack of probing in these areas during group interviews. In addition, there were other strategies that were discussed but could not be included in this paper. Instead, our analysis focused on the most frequently utilized strategies across the 10 sites. Despite the limitations this paper provides important insight on strategies for implementing CHWs in HIV care.

## Conclusion

Drawing on data from interviews with implementation teams, we examine CHW integration in the delivery of HIV care at ten Ryan White HIV/AIDS Program (RWHAP) funded recipient settings. Findings were consistent with the literature on implementing CHWs in ambulatory care sites. Factors that facilitated implementation included revising, defining, and differentiating professional roles and developing organizational policies and SOPs to support the role. Team meetings and huddles as well as other opportunities to support network weaving in addition to supportive and clinical supervision were also critical to CHW integration. Finally, adaptability as well as ongoing training and TA contributed to CHW integration.

## Data Availability

The codebook is available upon request. Contact: Linda Sprague Martinez, lsmarti@bu.edu

## Abbreviations

CHW: Community health worker
ERIC: Expert recommendations for implementing change
MSW: Master of social work
IRB: Institutional review board
PLWA: People lining with AIDS
RWAP: Ryan white HIV/AIDS program
SOP: Standard operating procedures
TA: Technical assistance
